# Survival of advanced/recurrent gastrointestinal stromal tumors treated with tyrosine kinase inhibitors in Taiwan: a nationwide registry study

**DOI:** 10.1186/s12885-024-12567-1

**Published:** 2024-07-11

**Authors:** Hui-Jen Tsai, Yan-Shen Shan, Ching-Yao Yang, Chin-Fu Hsiao, Chung-Hsin Tsai, Chuan-Cheng Wang, Ming-Tsan Lin, Chun-Fu Ting, De-Chuan Chan, Te-Hung Chen, Chueh-Chuan Yen, Yen-Yang Chen, Hsuan-Yu Lin, Ta-Sen Yeh, Ching-Liang Ho, Tze-Yu Shieh, Li-Yaun Bai, Jun-Te Hsu, I-Shu Chen, Li-Tzong Chen, Chun-Nan Yeh, Taiwan Cooperative Oncology Group (TCOG) GIST Study Group, Tsang-Wu Liu, Chieh-Han Chuang, Tsang-En Wang

**Affiliations:** 1 National Institute of Cancer Research, National Health Research Institutes, Tainan, Taiwan; 2 Department of Oncology, National Cheng Kung University Hospital, College of Medicine, National Cheng Kung University, Tainan, Taiwan; 3 Division of Hematology and Oncology, Department of Internal Medicine, Kaohsiung Medical University Hospital, Kaohsiung Medical University, Kaohsiung, Taiwan; 4 Department of Surgery, National Cheng Kung University Hospital, College of Medicine, National Cheng Kung University, Tainan, Taiwan; 5 Department of Surgery, National Taiwan University Hospital, National Taiwan University College of Medicine, Taipei, Taiwan; 6 Institute of Population Health Sciences, National Health Research Institutes, Zhunan, Taiwan; 7 Department of Surgery, MacKay Memorial Hospital and Mackay Medical College, Taipei, Taiwan; 8 Department of Internal Medicine, Changhua Christian Hospital, Changhua, Taiwan; 9 Department of Internal Medicine, China Medical University Hospital, Taichung, Taiwan; 10 Division of General Surgery, Department of Surgery, Tri-Service General Hospital, National Defense Medical Center, Taipei, Taiwan; 11 Department of Surgery, China Medical University Hospital, China Medical University, Taichung, Taiwan; 12 Division of Medical Oncology, Center for Immuno-Oncology, Department of Oncology, Taipei Veterans General Hospital, Taipei, Taiwan; 13 Division of Clinical Research, Department of Medical Research, Taipei Veterans General Hospital, Taipei, Taiwan; 14 School of Medicine, College of Medicine, National Yang Ming Chiao Tung University, Taipei, Taiwan; 15 Institute of Biopharmaceutical Sciences, College of Pharmaceutical Sciences, National Yang Ming Chiao Tung University, Taipei, Taiwan; 16 Kaohsiung Chang Gung Memorial Hospital and Chang Gung University College of Medicine, Kaohsiung, Taiwan; 17 Division of General Surgery, Department of Surgery, Chang Gung Memorial Hospital, Linkou, Chang Gung University, Taoyuan, Taiwan; 18 Division of Hematology and Oncology, Department of Internal Medicine, Tri-Service General Hospital, National Defense Medical Center, Taipei, Taiwan; 19 Division of Hematology and Oncology, Medical Department, Taipei Tzu Chi Hospital, Taipei, Taiwan; 20 Division of Gastroenterology, Department of Internal Medicine, Mackay Memorial Hospital, Taipei, Taiwan; 21 Division of General Surgery, Department of Surgery, Kaohsiung Veterans General Hospital and National Yang Ming Chiao Tung University, Kaohsiung, Taiwan; 22 Center for Cancer Research, Kaohsiung Medical University, Kaohsiung, Taiwan; 23 Division of Gastroenterology, Department of Internal Medicine, Kaohsiung Medical University Hospital, Kaohsiung, Taiwan; 24 Institute of Stem Cell and Translational Cancer Research, Chang Gung Memorial Hospital at Linkou, Chang Gung University, Taoyuan, Taiwan; 25 School of Medicine, National Tsing Hua University, Hsinchu, Taiwan

**Keywords:** Gastrointestinal stromal tumor, Metastatic, Recurrent, Tyrosine kinase inhibitor, Registry study

## Abstract

**Background:**

Most gastrointestinal stromal tumors (GISTs) harbor *c-KIT* or *PDGFRA* mutations. Administration of tyrosine kinase inhibitors (TKIs) has significantly improved the survival of patients with GISTs. We aimed to evaluate the clinical outcome of advanced or recurrent GIST patients in Taiwan.

**Methods:**

Patients diagnosed between 2010 and 2020 were enrolled. The collected data included baseline characteristics, treatment pattern, treatment outcome, genetic aberrations and survival status. Progression-free survival (PFS) and overall survival (OS) were analyzed and plotted with the Kaplan–Meier method. Cox regression analysis was used to analyze the prognostic factors of survival.

**Results:**

A total of 224 patients with advanced or recurrent GISTs treated with TKIs were enrolled. All patients received imatinib treatment. Ninety-three and 42 patients received sunitinib and regorafenib treatment, respectively. The 48-month PFS and OS rates for patients treated with imatinib were 50.5% and 79.5%, respectively. *c-KIT* exon 9 and *PDGFRA* mutations were prognostic factors for a poor PFS and *PDGFRA* mutation was a prognostic factor for a poor OS in patients treated with imatinib in multivariate Cox regression analysis. The median PFS of patients who received sunitinib treatment was 12.76 months (95% confidence interval (CI), 11.01–14.52). Patients with *c-KIT* exon 9 mutations had a longer PFS than those with other genetic aberrations. The median PFS of patients treated with regorafenib was 7.14 months (95% CI, 3.39–10.89).

**Conclusions:**

We present real-world clinical outcomes for advanced GIST patients treated with TKIs and identify mutational status as an independent prognostic factor for patient survival.

**Supplementary Information:**

The online version contains supplementary material available at 10.1186/s12885-024-12567-1.

## Background

Gastrointestinal stromal tumors (GISTs) are neoplasms arising from mesenchymal tissue of the GI tract. The most common sites of GISTs are the stomach and small intestine. Most GISTs can be managed with curative surgery followed by adjuvant imatinib treatment or not. However, approximately 40% of patients may develop metastasis [1]. Imatinib is a tyrosine kinase inhibitor and has been shown to inhibit KIT phosphorylation and cell proliferation of *c-KIT* mutated HMC-1 cells by Heinrich in 2000 [2]. In 2001, Joensuu et al. have reported that an advanced GIST patient with *c-KIT* exon 11 mutation, who progressed after multiple treatment, including surgery, chemotherapy, thalidomide and interferon alfa, had a good response to imatinib treatment [3]. After that, imatinib has been shown to induce an overall response rate of approximately 45–68% and a median progression-free survival (PFS) and overall survival (OS) of 18–26 and 51–57 months, respectively, according to randomized phase II and III clinical trials [4–6]. Imatinib has become the standard first-line therapy for advanced GISTs. Many prognostic factors, such as sex, tumor size, mitotic count, Eastern Cooperative Oncology Group performance status (ECOG PS), neutrophil count, albumin level, and genetic alterations, have been reported to be associated with the outcome of imatinib treatment for advanced GISTs [6–9].

GIST patients may develop resistance to imatinib; however, sunitinib has been approved as a second-line treatment and is associated with a PFS of 6.8 months for advanced GIST patients who are intolerant to or in whom imatinib failed [10]. Moreover, regorafenib was approved in 2013 as a third-line treatment for advanced GIST patients who are intolerant to imatinib or sunitinib or in whom these treatments failed [11]. Both sunitnib and regorafenib are multi-targeted tyrosine kinase inhibitors. They competitively inhibit ATP-binding sites of several receptor tyrosine kinases, including KIT [12]. There are also prognostic factors reported for sunitinib treatment in advanced GIST patients, such as the neutrophil to lymphocyte ratio (NLR), neutrophil and platelet count and genotype [13–16]. However, the results of these studies are controversial. Similarly, performance status, biological factors (NLR, platelet to lymphocyte ratio), and genotype have also been reported to be associated with the outcome of regorafenib treatment in advanced GIST patients [17, 18]. Regarding genotype of GIST, around 75% of GISTs harbor *c-KIT* mutations. Around 10% to 20% of GISTs harbor *PDGFRA* and around 5% to 10% of GISTs do not harbor *c-KIT* or *PDGFRA* mutation. The genotype of GISTs is associated with clinical features and sensitivities to tyrosine kinase inhibitors [8, 19–22]. For example, *PDGFRA* mutation is more associated gastric location whereas *c-KIT* exon 9 mutation is more associated with intestinal location [19]. GIST patients with *c-KIT* exon 9 mutation is associated with lower response rate to imatinib than those with *c-KIT* exon 11 mutation and higher dose of imatinib is suggested for GIST patients harboring *c-KIT* exon 9 mutation [19, 22]. Imatinib, sunitinib and regorafenib have been approved and reimbursed by the health bureaus in Taiwan since 2002, 2010, and 2016, respectively. Thus, we conducted a registry study of GIST patients to understand the demographics, genotypes, treatment patterns, and treatment outcomes of these patients in Taiwan. In this registry study, we analyzed the baseline characteristics, treatment outcomes and prognostic factors for recurrent or metastatic GISTs in patients who received tyrosine kinase inhibitor (TKI) treatment.

## Methods

### Patients, study design and data collection

This was a longitudinal multicenter registry study (Taiwan Cooperative Oncology Group T1218). Eleven hospitals located from the northern to southern Taiwan, including Taiwan University Hospital, Taipei Veterans General Hospital, Mackay Memorial Hospital, Tri-Service General Hospital, Linkou Chang Gung Memorial Hospital, China Medical University Hospital, Changhua Christian Hospital, National Cheng Kung University Hospital, Kaohsiung Medical University Hospital, Kaohsiung Veterans General Hospital, and Kaohsiung Chang Gung Memorial Hospital, participated in this study. Pathologically proven GIST patients diagnosed between January 1, 2010, and December 31, 2020, were included. The data for enrolled patients were collected via chart review. The collected data included baseline characteristics, treatment strategies (surgery, TKI treatment, and any other therapy), genetic profiles, best treatment responses, PFS, OS and adverse events associated with TKI therapy. We retrospectively collected the data prior to enrollment and prospectively collected the data after enrollment. The study protocol was reviewed and approved by the Institutional Review Board of each participating institution. All patients except those who had died signed informed consent. In the current study, we analyzed the clinical outcomes of patients who received TKI treatment. Patients who received TKIs (imatinib, sunitinib and regorafenib) for less than 30 days were excluded from the analysis.

### Genetic analysis of GIST patients

Some patients had *c-KIT* and *PDGFRA* aberrations detected prior to enrollment. The data were recorded in our database system. Some patients did not undergo a check for *c-KIT* and/or *PDGFRA* before enrollment. Their tumor samples were sent to our central laboratory for *c-KIT* and *PDGFRA* assessment by Sanger sequencing. The detailed methods of DNA extraction, PCR, and Sanger sequencing, and the primer sequences for *c-KIT* and *PDGFRA* were described in Supplementary Method. Wild-type *c-KIT* and *PDGFRA* were defined as no aberration detected for *c-KIT* exons 9, 11, 13, 14 and 17 and *PDGFRA* exons 12, 14, and 18 and was called “wild-type *c-KIT/PDGFRA*” in this study. Ninety-five percent of tumor samples used for assessment of *c-KIT* and *PDGFRA* were free of TKI treatment.

### Statistical analysis

All statistical analyses were performed using SAS statistical software (Version 9.4, SAS Institute Inc., Cary, NC, U.S.A.). Descriptive analyses were performed to examine the baseline characteristics and genetic alterations of patients. Data were summarized using descriptive statistics (number of patients, mean, standard deviation, median, minimum, and maximum) for continuous variables and using frequency and percentage for categorical variables. Fisher’s exact test was used to compare the differences in baseline characteristics for categorical variables, while t-test was performed for continuous variables. If the normality assumption for a continuous endpoint was violated, the nonparametric Wilcoxon–Mann–Whitney test was applied. PFS was defined as the time from the date of TKI use (imatinib, sunitinib, or regorafenib) to the date of disease progression. OS was defined as the time from the date of TKI use to the date of patient death due to any cause or to the last date that the patient was known to be alive. Probabilities of PFS and OS were estimated by the method of Kaplan–Meier. Univariate Cox proportional hazards model was used to evaluate the risk associated with baseline characteristics for the PFS and OS. Baseline variables with significant difference from the univariate analyses were selected in multivariate analysis. Hazard ratios and 95% confidence intervals were also calculated. All tests were two-tailed. A *P* value < 0.05 was considered significant.

## Results

### Demographics of advanced/metastatic or recurrent GIST patients who received imatinib treatment

There were 224 patients who received imatinib treatment due to a diagnosis of advanced/metastatic disease or recurrent disease after prior surgery with or without adjuvant imatinib therapy. They were classified into 4 groups by the timing of imatinib use for controlling advanced/metastatic or recurrent disease but not for adjuvant purpose. Eighty-nine patients were treated with primary front-line imatinib. Seventy-six patients were treated with imatinib due to recurrent disease after surgery with adjuvant imatinib. Twenty-seven patients were treated with imatinib due to recurrent disease after prior surgery without adjuvant imatinib. Thirty-two patients were treated with imatinib after palliative surgery. The male to female ratio was 1.43. The median age of all 224 patients was 57.8 years (19.6 to 85.1). The ECOG PS scores among the 121 patients with known ECOG PS were 0 for 55 patients and 1 for 55 patients. Eighty-five (37.9%) patients had GISTs of the stomach. Ninety-seven (43.3%) patients had GISTs of the small intestine, which included duodenum, jejunum, ileum, and other small intestine with 20, 43, 21, and 13 patients, respectively. The other primary sites of GISTs included the esophagus, colon, rectum, peritoneum, vagina, prostate and one undetermined site. The baseline characteristics, including stage, of these 224 patients by primary site of the stomach and nonstomach are shown in Table 1. The known genetic data among the patients are also listed in Table 1. One hundred thirty-six (76.8%) patients had *c-KIT* exon 11 mutations with mutational type of deletion, missense mutation, deletion + missense mutation, deletion and insertion, and duplication. Eighteen (10.2%) patients had *c-KIT* exon 9 mutations. Seventeen of the 18 patients had 502–503 duplication mutation and the other one had insertion mutation. Three (1.7%) patients had *PDGFRA* mutations. Two of the 3 patients had D842V mutation and the other one had V561D mutation. Seventeen (9.6%) patients had wild-type *c-KIT/PDGFRA*. The percentages of the baseline albumin level and NLR in the patients are also listed in Table 1.

**Table 1:** baseline characteristics of the patients who received imatinib treatment

	Stomach	Nonstomach	Overall	P* value
Timing of imatinib use	85	139	224	< 0.0001
primary front-line imatinib	47 (55.3%)	42 (30.2%)	89 (39.7%)	
recurrence (with prior adjuvant imatinib)	26 (30.6%)	50 (36.0%)	76 (33.9%)	
recurrence (without prior adjuvant imatinib)	9 (10.6%)	18 (12.9%)	27 (12.1%)	
Imatinib after palliative surgery	3 (3.5%)	29 (20.9%)	32 (14.3%)	
Sex	85	139	224	0.5781
men	48 (56.5%)	84 (60.4%)	132 (58.9%)	
women	37 (43.5%)	55 (39.6%)	92 (41.1%)	
mean age (± std)	60.2 (± 12.7)	56.8 (± 12.8)	58.1 (± 12.8)	0.0586^#^
min	32.4	19.6	19.6	
median	60.0	55.5	57.8	
max	85.1	84.5	85.1	
ECOG PS	40	81	121	0.3924
0	19 (47.5%)	36 (44.4%)	55 (45.5%)	
1	18 (45.0%)	37 (45.7%)	55 (45.5%)	
2	1 (2.5%)	7 (8.6%)	8 (6.6%)	
3	2 (5.0%)	1 (1.2%)	3 (2.5%)	
Stage	81	132	213	0.2149
I	8 (9.9%)	7 (5.3%)	15 (7.0%)	
II	10 (12.3%)	18 (13.6%)	28 (13.1%)	
III	24 (29.6%)	55 (41.7%)	79 (37.1%)	
IV	39 (48.1%)	52 (39.4%)	91 (42.7%)	
Primary site	85	139	224	< 0.0001
esophagus	0	6 (4.3%)	6 (2.7%)	
stomach	85 (100%)	0	85 (37.9%)	
duodenum	0	20 (14.4%)	20 (8.9%)	
jejunum	0	43 (30.9%)	43 (19.2%)	
ileum	0	21 (15.1%)	21 (9.4%)	
small intestine, others	0	13 (9.4%)	13 (5.8%)	
colon	0	6 (4.3%)	6 (2.7%)	
rectum	0	14 (10.1%)	14 (6.3%)	
peritoneum (include omentum)	0	13 (9.4%)	13 (5.8%)	
others^a^	0	3 (2.2%)	3 (1.3%)	
Genetic alteration	65	112	177	0.0014
* c-KIT* exon 9	1 (1.5%)	17 (15.2%)	18 (10.2%)	
* c-KIT* exon 11	55 (84.6%)	81 (72.3%)	136 (76.8%)	
* c-KIT* exon 13	1 (1.5%)	0	1 (0.6%)	
* c-KIT* exon 17	2 (3.1%)	0	2 (1.1%)	
* PDGFRA*	2 (3.1%)	1 (0.9%)	3 (1.7%)	
Wild-type *c-KIT/ PDGFRA*	4 (6.2%)	13 (11.6%)	17 (9.6%)	
Baseline albumin level	47	83	130	0.0088
< 3.2 g/dl	14 (29.8%)	9 (10.8%)	23 (17.7%)	
≥ 3.2 g/dl	33 (70.2%)	74 (89.2%)	107 (82.3%)	
Baseline neutrophil/lymphocyte ratio	70	121	191	0.6460
< 3.0	26 (37.1%)	50 (41.3%)	76 (39.8%)	
≥ 3.0	44 (62.9%)	71 (58.7%)	115 (60.2%)	

^a^one vagina, one prostate and one undetermined

^*^Fisher’s exact test

^#^t-test

### Genetic mutation profiles among advanced/metastatic or recurrent GIST patients who received TKI treatment

We analyzed the genetic alterations in patients to understand whether these alterations were different according to the baseline patient characteristics (Table 2). Higher percentage of male patients had *c-KIT* exon 9 mutations (men vs women, 72.2% vs 27.8%), *PDGFRA* mutations (men vs women, 100.0% vs 0%), and wild-type *c-KIT/PDGFRA* (men vs women, 82.4% vs 17.6%) than female patients (*P* = 0.0215). Higher percentage of nonstomach GIST patients had *c-KIT* exon 9 mutations (nonstomach vs stomach, 94.4% vs 5.6%) and wild-type *c-KIT/PDGFRA* (nonstomach vs stomach, 76.5% vs 23.5%) than stomach GIST patients (*P* = 0.01). Higher percentage of patients aged less than 60 years had *c-KIT* exon 9 mutations (< 60 years vs ≥ 60 years, 83.3% vs 16.7%) and wild-type *c-KIT/PDGFRA* (< 60 years vs ≥ 60 years, 76.5% vs 23.5%) than those aged more than 60 years (*P* = 0.0205). The genetic alterations were not significantly different among patients with different ECOG PS scores.

**Table 2:** Genetic alterations in advanced/metastatic or recurrent GIST patients who received TKI treatment

Genotype	*c-KIT* exon 9	*c-KIT* exon 11	*c-KIT* exon 13	*c-KIT* exon 17	*PDGFRA*	Wild-type *c-KIT/PDGFRA*	overall	*P* * value
Sex	18	136	1	2	3	17	177	0.0215
men	13(72.2%)	71(52.2%)	1(100.0%)	2 (100.0%)	3 (100.0%)	14 (82.4%)	104 (58.8%)	
women	5 (27.8%)	65 (47.8%)	0	0	0	3 (17.6%)	73 (41.2%)	
Primary site	18	136	1	2	3	17	177	0.01
stomach	1 (5.6%)	55 (40.4%)	1 (100.0%)	2 (100.0%)	2 (66.7%)	4 (23.5%)	65 (36.7%)	
nonstomach	17 (94.4%)	81 (59.6%)	0	0	1 (33.3%)	13 (76.5%)	112 (63.3%)	
ECOG PS	12	75	1	0	1	9	98	0.5927
0	4 (33.3%)	34 (45.3%)	0	0	0	5 (55.6%)	43 (43.9%)	
1	8 (66.7%)	33 (44.0%)	1 (100.0%)	0	1 (100.0%)	3 (33.3%)	46 (46.9%)	
2	0	6 (8.0%)	0	0	0	0	6 (6.1%)	
3	0	2 (2.7%)	0	0	0	1 (11.1%)	3 (3.1%)	
Age	18	136	1	2	3	17	177	0.0205
< 60	15 (83.3%)	74 (54.4%)	0	2 (100.0%)	1 (33.3%)	13 (76.5%)	105 (59.3%)	
> = 60	3 (16.7%)	62 (45.6%)	1 (100.0%)	0	2 (66.7%)	4 (23.5%)	72 (40.7%)	

^*^Fisher’s exact test

### PFS and OS of advanced/metastatic or recurrent GIST patients who received imatinib treatment

**Fig. 1:**
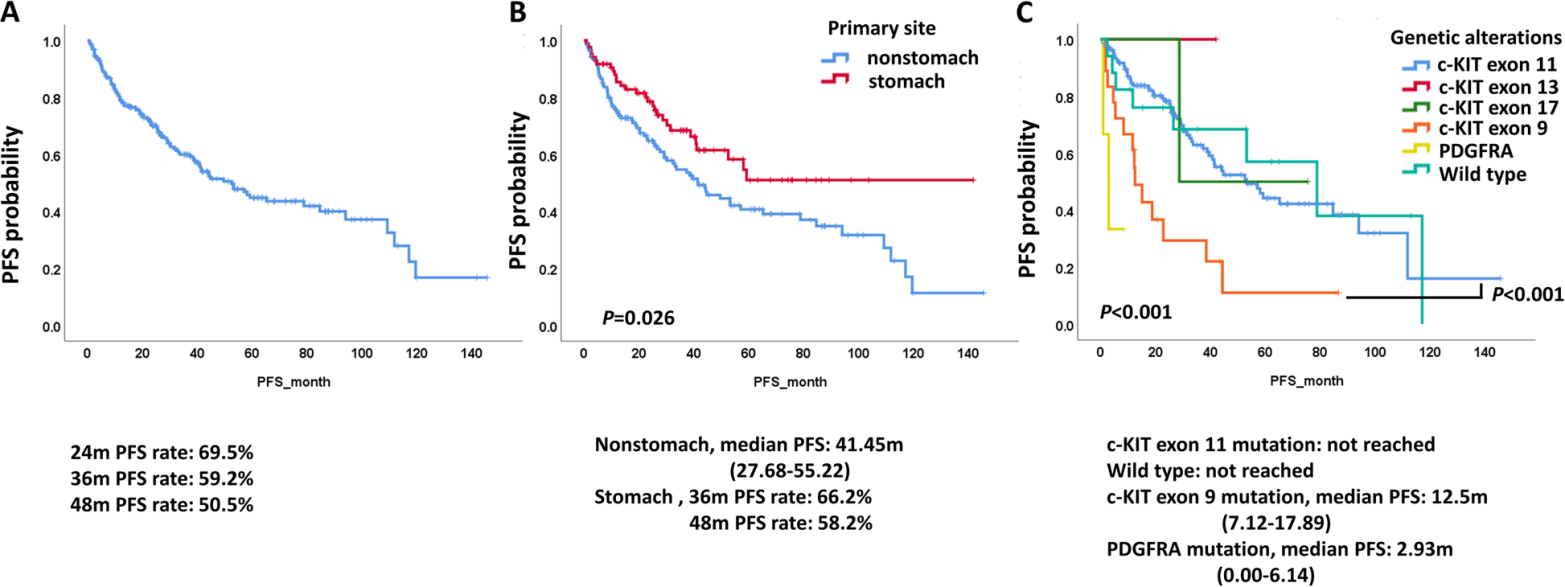
PFS of advanced or recurrent GIST patients who received imatinib treatment. **A** PFS of all GIST patients. **B** PFS of GIST patients by primary site. **C** PFS of GIST patients by genetic alterations

We analyzed the PFS of 224 advanced/metastatic or recurrent GIST patients who received imatinib treatment in our registry study. The median PFS was not reached, and the 48-month PFS rate was 50.5% (Fig. 1A). We investigated whether the PFS or OS was different among patients with initial advanced/metastatic disease with or without palliative surgery or recurrent disease after prior surgery with or without adjuvant imatinib treatment. The PFS among these 4 groups was not significantly different, as shown in Supplementary Fig. 1. The PFS analysis in patients was also not significantly different by sex, ECOG PS score, baseline albumin level, and baseline NLR, as shown in Supplementary Fig. 2. The median PFS of all patients is shown in Fig. 1A. The median PFS was significantly different in patients who received imatinib by primary site and genetic profiles. The median PFS of patients with a primary site of the stomach was not reached, and the median PFS of patients with a nonstomach primary site was 41.45 (95% confidence interval (CI), 27.68–55.22) months (Fig. 1B) (*P* = 0.026). Patients with *c-KIT* exon 11 mutations (not reached) and wild-type *c-KIT/PDGFRA* (not reached) had a longer PFS than patients with *c-KIT* exon 9 (median 12.5 (95% CI, 7.12–17.89) months) and *PDGFRA* mutations (median 2.93 (95% CI, 0.00–6.14) months) (*P* < 0.001) (Fig. 1C).

**Fig. 2:**
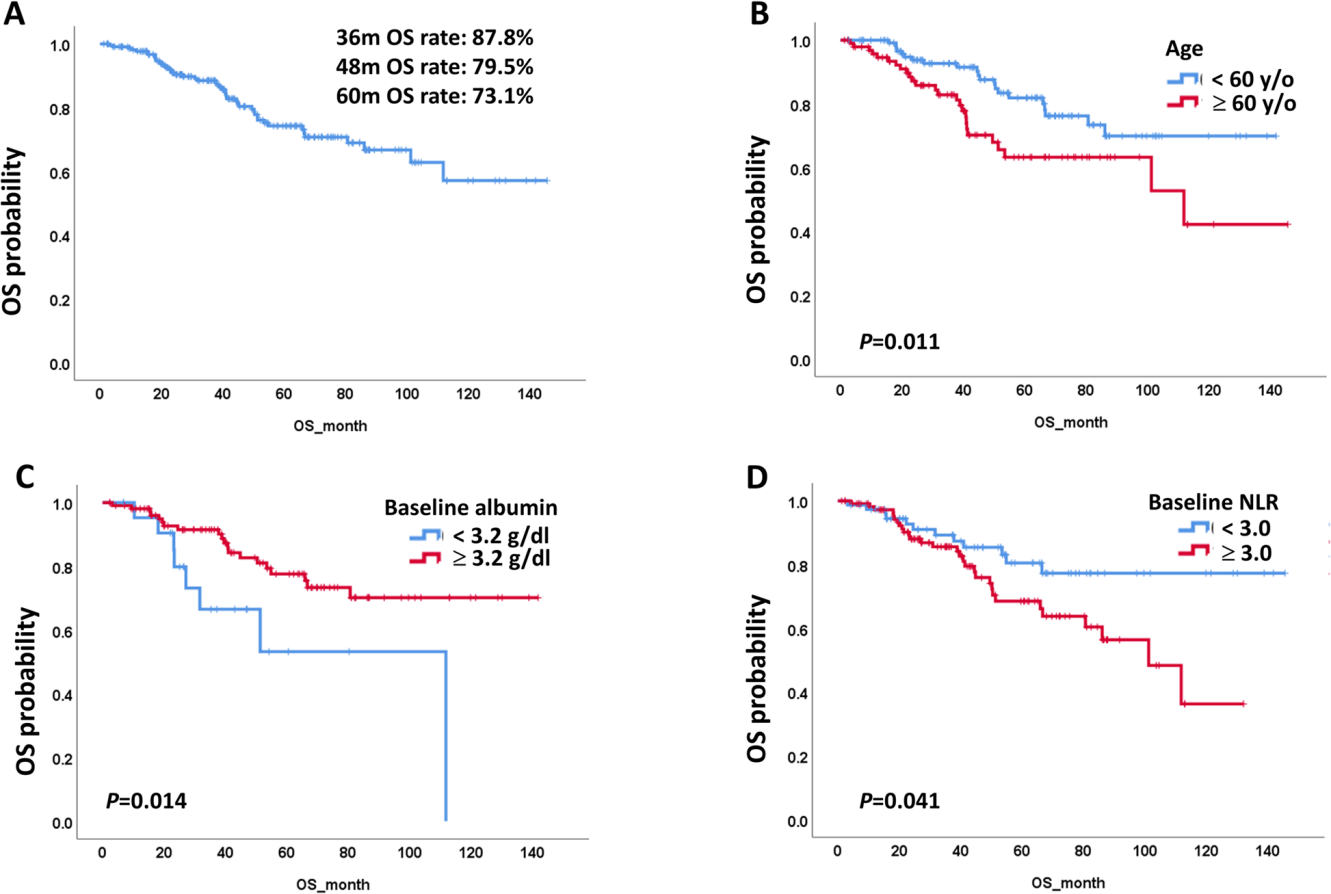
OS of advanced or recurrent GIST patients who received imatinib treatment. **A** OS of all GIST patients. **B** OS of GIST patients by age. **C** OS of GIST patients by baseline albumin level. **D** OS of GIST patients by baseline NLR

The median OS was not reached, and the 48-month OS rate was 79.5% (Fig. 2A). The OS among the 4 groups of patients who received imatinib was not significantly different. The OS was also not significantly different in patients according to sex, ECOG PS score, primary site, and genetic alterations, as shown in Supplementary Fig. 3. Regarding genetic alterations in patients, the OS among all groups with different genetic alterations was not significantly different. Patients with *PDGFRA* mutations had a significantly shorter OS (median OS 18.00 (95% CI, 4.10–31.89) months) than patients with other genetic alterations. The OS of patients with *c-KIT* exon 9 mutations was not significantly different from that of patients with *c-KIT* exon 11 mutations. The OS of all patients is shown in Fig. 2A. The OS was significantly different in patients according to age (Fig. 2B), baseline albumin level (Fig. 2C) and baseline NLR (Fig. 2D). The OS of patients aged ≥ 60 years was worse than that of patients aged < 60 years (*P* = 0.011). The OS of patients with a baseline albumin level < 3.2 g/dl was worse than that of patients with a baseline albumin level ≥ 3.2 g/dl (*P* = 0.014). The OS of patients with a baseline NLR ≥ 3.0 was worse than that of patients with a baseline NLR < 3.0 (*P* = 0.041).

### Univariate and multivariable Cox regression analysis of PFS and OS in advanced/metastatic or recurrent GIST patients who received imatinib treatment

We performed univariate and multivariable Cox regression analyses to evaluate the risk associated with baseline characteristics for the PFS of these patients. Supplementary Table 1 shows the univariate Cox regression analysis for the PFS of these patients by sex, age, ECOG PS score, primary site, baseline albumin level, baseline NLR and genetic alterations. In univariate analysis, patients with mutations in *c-KIT* exon 9 (hazard ratio (HR) = 3.057, 95% CI, 1.694–5.519, *P* = 0.0002) or *PDGFRA* (HR = 13.178, 95% CI, 3.015–57.597, *P* = 0.0006) had a higher HR than patients with mutations in *c-KIT* exon 11. Patients with a primary site in the stomach had a lower risk (HR = 0.617, 95% CI, 0.401–0.949, *P* = 0.0280) than those with a nonstomach primary site. In multivariable analysis (Table 3), only *c-KIT* exon 9 (HR = 2.997, 95% CI, 1.620–5.544, *P* = 0.0005) and *PDGFRA* mutations (HR = 13.609, 95% CI, 3.029–61.138, *P* = 0.0007) were still statistically significant, with a higher HR for PFS in these patients.

**Table 3:** Multivariate Cox regression analysis for PFS of recurrent/metastatic GIST patients treated with imatinib treatment

Multivariate analysis			
	HR	95% CI	*P* value
*c-KIT* exon 13	-	-	-
*c-KIT* exon 17	0.889	0.120–6.609	0.9085
*c-KIT* exon 9	2.997	1.620–5.544	0.0005
*PDGFRA*	13.609	3.029–61.138	0.0007
Wild-type *c-KIT/PDGFRA*	0.876	0.409–1.877	0.7328
Primary site, referent nonstomach			
stomach	0.943	0.563–1.580	0.8228

The univariate Cox regression analysis for the OS of these patients by sex, age, ECOG PS score, primary site, baseline albumin level, baseline NLR and genetic alterations is shown in Supplementary Table 2. *PDGFRA* mutation (HR = 4.815, 95% CI, 1.140–20.345, *P* = 0.0325), age ≥ 60 years (HR = 2.074, 95% CI, 1.164–3.694, *P* = 0.0133), and baseline NLR ≥ 3.0 (HR = 1.987, 95% CI, 1.014–3.895, *P* = 0.0454) were risk factors for poor OS in patients. Baseline albumin level ≥ 3.2 g/dl (HR = 0.365, 95% CI, 0.158–0.841, *P* = 0.0180) was a favorable factor for OS in these patients. In multivariate analysis, only *PDGFRA* mutation (HR = 98.670, 95% CI, 5.200–1872.32, *P* = 0.0022) was a risk factor for poor OS in these patients (Supplementary Table 3).

### c-KIT exon 11 mutation and the impact of exon 11 mutational type on the PFS and OS of advanced/metastatic or recurrent GIST patients who received imatinib treatment

We analyzed the mutational type patterns of the 136 recurrent or advanced/metastatic GIST patients with *c-KIT* exon 11 mutation who received imatinib treatment (Supplementary Table 4). The number of patients with *c-KIT* exon 11 deletion, missense mutation, deletion + missense mutation, deletion and insertion, and duplication was 69, 24, 32, 8, and 2, respectively. One patient’s mutation type was unknown. There was no difference in the percentage of mutational type between GISTs of the stomach or nonstomach (*P* = 0.2263). The PFS of patients with the 3 major types (deletion, missense mutation, deletion + missense mutation) of *c-KIT* exon 11 mutations is shown in Supplementary Fig. 4A. The PFS of patients with deletions was better than that of patients with deletions + missense mutations and missense mutations in *c-KIT* exon 11 (*P* = 0.023). The median PFS of patients with deletion and deletion + missense mutations in *c-KIT* exon 11 was not reached. The median PFS of patients with *c-KIT* exon 11 missense mutations was 33.13 (95% CI, 14.41–51.84) months. Supplementary Fig. 4B shows the OS of patients with the 3 major types of mutations in *c-KIT* exon 11. The OS of patients with deletions was better than that of patients with deletions + missense mutations and missense mutations in *c-KIT* exon 11 (*P* = 0.004).

### PFS and OS of advanced/metastatic or recurrent GIST patients who received sunitinib treatment

**Fig. 3:**
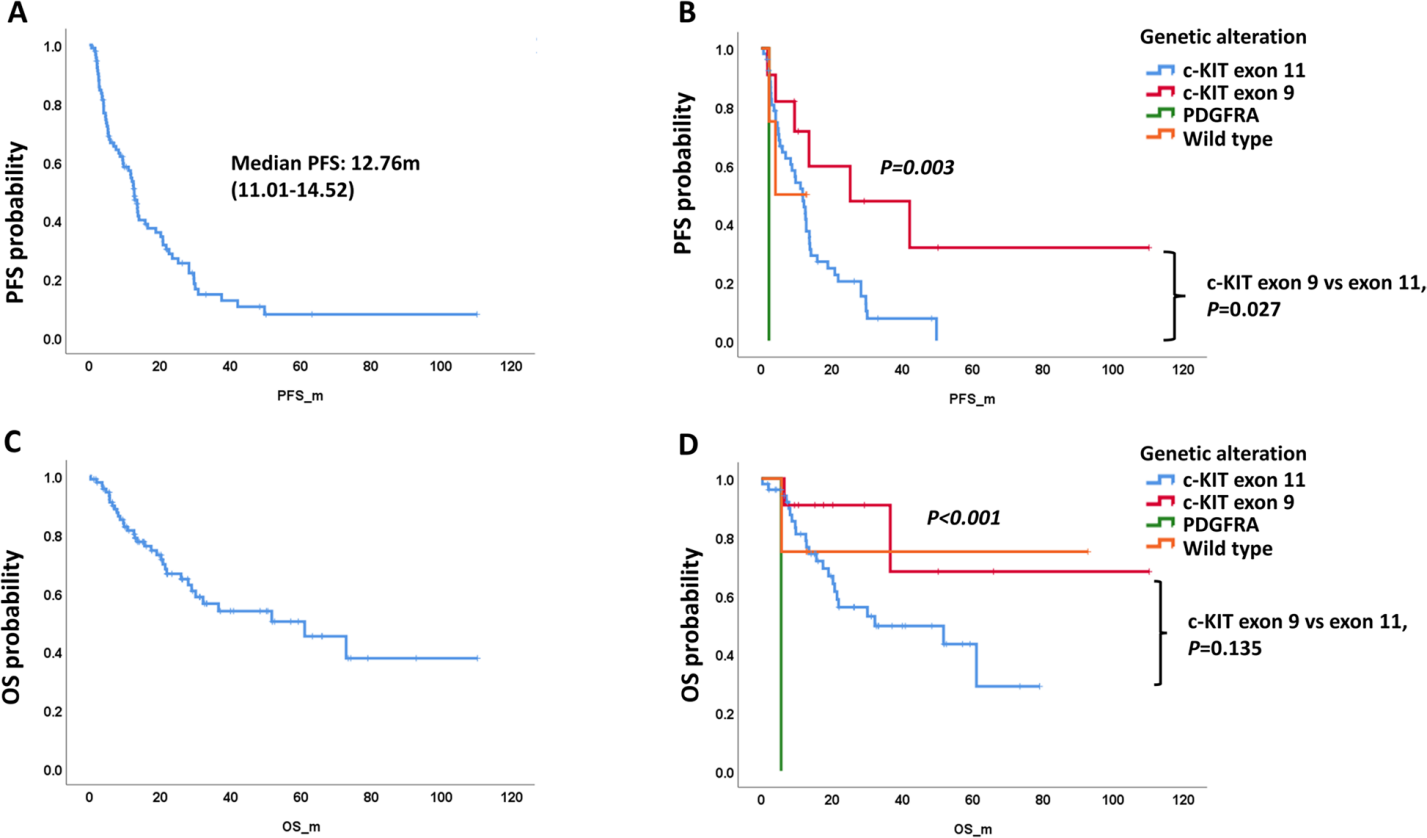
PFS and OS of advanced or recurrent GIST patients who received sunitinib treatment. **A** Median PFS of all patients. **B** PFS of GIST patients by genetic alterations. **C** Median OS of all patients. **D** OS of GIST patients by genetic alterations

Most of the patients who were intolerant to or in whom imatinib treatment failed were treated with sunitinib. We analyzed the PFS and OS of 93 patients who received sunitinib treatment after imatinib failure. The baseline characteristics of these patients are listed in Supplementary Table 5. Sixty-five patients had nonstomach GISTs, and the other 28 patients had stomach GISTs. There was no difference in the distribution of sex and ECOG PS score between patients with stomach and nonstomach GISTs. However, only one stomach GIST patient but 10 nonstomach GIST patients had *c-KIT* exon 9 mutations. The median PFS of all patients treated with sunitinib was 12.76 (95% CI, 11.01–14.52) months, as shown in Fig. 3A. There was no difference in PFS in patients by sex, ECOG PS score, and primary site (Supplementary Fig. 5). However, the PFS was different among patients with different genetic alterations. Patients with *c-KIT* exon 9 mutations had a longer PFS than those with *c-KIT* exon 11 mutation, *PDGFRA* mutation and wild-type *c-KIT/PDGFRA* (*P* = 0.003), as shown in Fig. 3B. The median PFS of the patient with *c-KIT* exon 9 mutation was 25.26 months, whereas the median PFS values for patients with *c-KIT* exon 11 mutation, *PDGFRA* mutation, and wild-type *c-KIT/PDGFRA* were 11.74, 2.17, and 4.01 months, respectively. The median OS of the 93 patients was not reached, with 36-month and 60-month survival rates of 53.8% and 45.2%, respectively (Fig. 3C). The OS was not significantly different in patients by sex, ECOG PS score, and primary site (Supplementary Fig. 5). The OS was different in patients by genetic alterations, with a longer OS in patients with *c-KIT* exon 9 mutation than in those with *PDGFRA* mutation and *c-KIT* exon 11 mutation (P < 0.001). However, the OS was not significantly different between patients with *c-KIT* exon 9 and exon 11 mutations (*P* = 0.135) (Fig. 3D).

### PFS and OS of advanced/metastatic or recurrent GIST patients who received regorafenib treatment

**Fig. 4:**
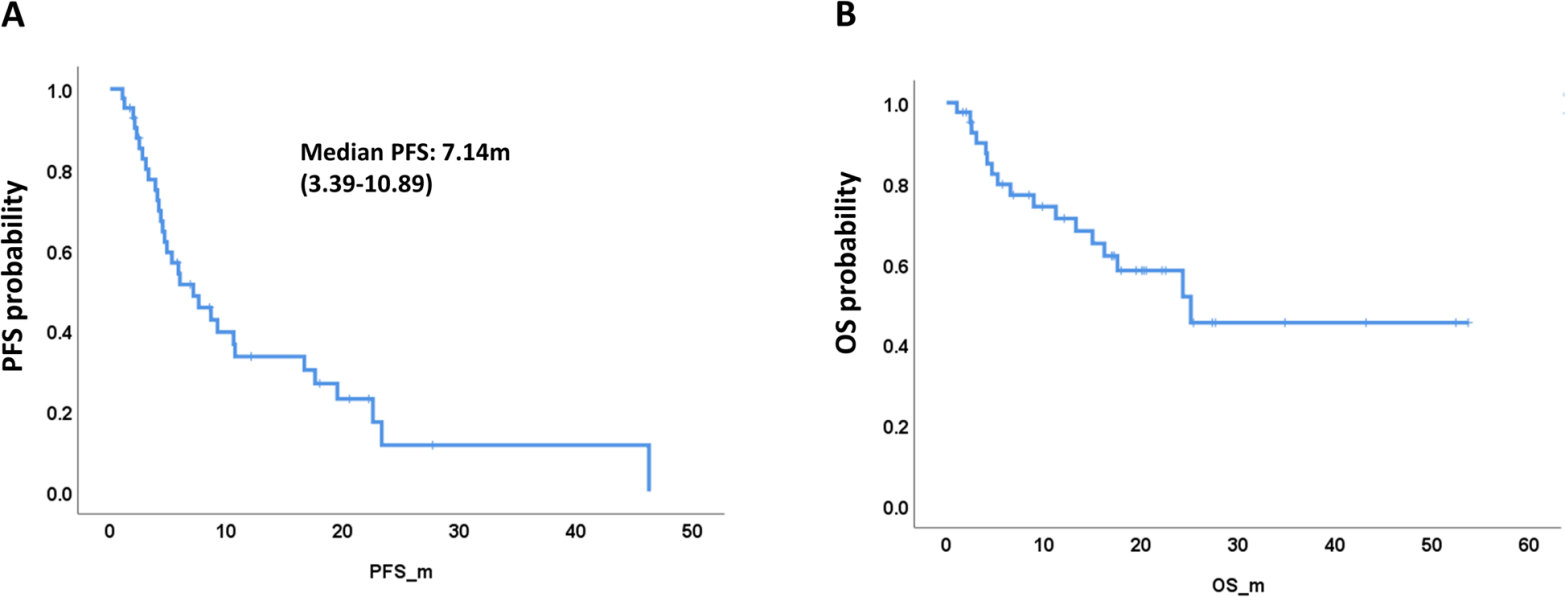
PFS and OS of advanced or recurrent GIST patients who received regorafenib treatment. **A** PFS of all patients. **B** OS of all patients

The patients who were intolerant to or in whom imatinib and sunitinib failed received regorafenib treatment. We analyzed the PFS and OS of the 42 patients who received regorafenib treatment. The median PFS of patients treated with regorafenib was 7.14 (95% CI, 3.39–10.89) months (Fig. 4A). There was no difference in PFS by primary site, sex or ECOG PS score (Supplementary Fig. 6). The median OS was not reached, with 12-month and 24-month survival rates of 68.1% and 51.8%, respectively (Fig. 4B). There was no significant difference in OS by primary site, sex, or ECOG PS score (Supplementary Fig. 6). Because only one case had a *PDGFRA* mutation and one case had wild-type *c-KIT/PDGFRA*, these two cases were not included in the analysis for the effect of genetic alterations on PFS and OS. The difference in PFS and OS by *c-KIT* exon 9 and *c-KIT* exon 11 mutations was not significant in patients who received regorafenib treatment (Supplementary Fig. 7).

## Discussion

This study shows a longer PFS and OS in advanced and recurrent GIST patients who received TKI therapies diagnosed between 2010 and 2020 in Taiwan. Genetic aberrations are prognostic factors for PFS and OS in patients who received imatinib and sunitinib treatment.

Imatinib was approved by the Food and Drug Administration (FDA) for the treatment of advanced GISTs in 2001. The median PFS and OS of advanced GIST patients who received imatinib treatment were 18–26 and 51–57 months, respectively, according to registered clinical trials [4–6]. The survival of our patients was longer, with 48-month PFS and OS rates of patients treated with imatinib of 50.5% and 79.5%, respectively. The data from the Netherlands Cancer Registry show that the 5-year OS rate was 48.2% for patients with primary metastatic GIST diagnosed between 2001 and 2012 [23]. Data from the GOLD ReGISTry, a global prospective, observational registry study between 2007 and 2011, showed that the estimated 30-month PFS and OS rates were 59.8% and 82.7%, respectively, for 1095 advanced GIST patients [24]. Data from the Dutch GIST Registry show that the median PFS and OS of 420 advanced GIST patients diagnosed between 2009 and 2021 who were treated with imatinib were 33.0 and 68.0 months, respectively [25]. These real-world data all showed longer survival for advanced GIST patients. Less extensive disease and earlier treatment with imatinib were possible reasons for the longer survival in these data since the patients were diagnosed after the approval of imatinib. In addition, most physicians are familiar with the management of adverse events of imatinib, which affects patient compliance with the drug. On the other hand, sunitinib and regorafenib were approved by the FDA for the treatment of GISTs in 2006 and 2013, respectively. The use of imatinib, sunitinib and regorafenib has been reimbursed in Taiwan since 2002, 2010, and 2016, respectively. Sunitinib and regorafenib treatment were available as further treatment for the patients in whom imatinib and sunitinib failed after the approval date, which explains the longer OS of the patients than the previous registration trials.

In the current study, we showed the incidence of *c-KIT* and *PDGFRA* aberrations in advanced or recurrent GIST patients (Table 2). The percentages of genetic aberrations in *c-KIT* and *PDGFRA* and wild-type *c-KIT/PDGFRA* were 88.7%, 1.7% and 9.6%, respectively. The percentage of *PDGFRA* aberration reported in GISTs, including localized and advanced stage, was 10–16.3% [26–28]. Our data showed a much lower percentage of *PDGFRA* in advanced or recurrent GISTs, which is consistent with < 4% of *PDGFRA* mutations in advanced GISTs from the S0033 trial [9, 29]. Similar to other studies, most *c-KIT* exon 9 mutations were detected in nonstomach GISTs [26, 27]. In the current study, we found that men had more *c-KIT* exon 9 mutations than women, and the 3 patients with *PDGFRA* mutations were all men. Because the sample size was small, more data are needed for further confirmation.

In the current study, we analyzed the potential prognostic factors for PFS and OS in advanced or recurrent GIST patients treated with TKI therapy. For the patients treated with imatinib, genetic aberrations and primary site were prognostic factors for PFS in univariate Cox regression analysis. However, only *c-KIT* exon 9 and *PDGFRA* mutations were associated with a poor PFS compared with *c-KIT* exon 11 mutations in multivariate analysis. Genetic aberrations, age, baseline albumin level and baseline NLR were prognostic factors for OS in univariate analysis. However, only *PDGFRA* mutation was associated with a poor OS in multivariate analysis. Gold et al. reported that the mutational status of *c-KIT* and *PDGFRA* was not associated with the outcome of metastatic GIST patients before the use of TKIs [30]. *c-KIT* exon 11 mutation has been reported to be associated with a better OS than *c-KIT* exon 9 or wild-type advanced GIST treated with imatinib [8, 20]. In our current analysis, sunitinib was available for all patients in whom imatinib treatment failed. We also observed that patients with *c-KIT* exon 9 mutations had a longer PFS and OS under sunitinib treatment. Therefore, it is reasonable that the OS of patients with *c-KIT* exon 9 and exon 11 mutations was not significantly different in our current study. However, patients with *PDGFRA* mutations had the worst survival since this mutation is generally not responsive to imatinib, sunitinib or regorafenib [21]. In the current study, two patients had *PDGFRA* D842V mutation and the PFS of these two patients to front-line imatinib were 2.9 and 0.9 month, respectively. The first patient then received sunitinib and regorafenib with the PFS of 2.2 and 1.2 months, respectively. The second patient developed enlargement of huge tumor and massive ascites after approximately 1 month’s imatinib and then received surgical removal of tumor with suspected seeding tumor in liver. This patient continued imatinib treatment after surgery with stable disease for 73 months and then received sunitinib treatment with a PFS of 2.7 months. This patient then received avapretinib treatment and is still kept stable disease. Consistent with previous studies, our data showed shorter PFS for patients with *PDGFRA* D842V mutation treated with imatinib, sunitinib, and regorafenib. For the patients with *c-KIT* exon 11 mutations, Incorvaia et al. have reported that 60 metastatic GIST patients with their tumor harboring deletion or insertion/deletion in codons 557 and/or 558 (D-557/8) had shorter PFS to first-line imatinib than the patients with their tumors harboring mutations other than D-557/8 [31]. In our current study, because the data of deletion site were not available in some patients, the analysis for D-557/8 was not performed. We showed that the PFS of the patients with *c-KIT* exon 11 deletion was longer than those with *c-KIT* exon 11 deletion + missense mutation or missense mutation when they received first-line imatinib treatment. Our result is not consistent with Incorvaia et al.’s result. Although several study groups have reported the poor prognostic role of D-557/8 or deletion in *c-KIT* exon 11 on recurrence free survival in resected GIST patients, the impact of mutational type of *c-KIT* exon 11 on survival of advanced GIST patients needs more data for further confirmation [32–34]. The genetic study for *c-KIT* and *PDGFRA* is not routinely performed for GISTs because it is not reimbursed in Taiwan and the application of sunitinib or regorafenib is feasible without genetic data. Our result suggests that genetic test is strongly indicated for the patients experiencing resistance to imatinib, particularly early resistance due to *PDGFRA* exon 18 mutations which may benefit from novel TKI, avapretinib, therapy [35]. Regarding the other risk factors associated with the survival of advanced GIST patients treated with TKIs, such as ECOG PS score, age, sex, baseline neutrophil count, and baseline albumin level [5, 13, 20, 36], we could not identify their prognostic role after multivariate analysis.

There are some limitations of this study. The lack of patient data, particularly ECOG PS score and baseline albumin level, is a limitation of this study. Another limitation of this study is the patient selection bias that we enrolled the patients treated in the 11 medical centers but not in regional hospitals. The resources, availability of medications, and practical principles in regional hospitals may differ from that in medical centers and affect the survival of the cancer patients [37]. To overcome these problems, a prospective registry study with predefined baseline characteristics and biomarkers been evaluated and checked in GIST patients from medical centers and regional hospitals may provide more comprehensive information. However, previous studies were analyzed earlier, and imatinib was the major treatment for these patients [5, 13, 20, 36]. Our patient population had more treatment options, namely, sunitinib and regorafenib, after imatinib failure. Recently, novel TKIs, such as ripretinib and avapritinib, have been evaluated and approved for refractory advanced GIST or *PDGFRA* exon 18-mutated GIST patients by FDA in May 2020 and January 2020, respectively, based on the results of randomized phase III trials [35, 38]. The efficacy of other novel agents, such as the heat shock protein 90 inhibitor TAS-116, has also been evaluated in clinical trials [39, 40]. Therefore, we expect that the survival of advanced GIST patients will be longer and that the effect of mutational status will probably become less significant after the availability of effective novel agents for GIST treatment in the near future.

## Conclusions

Our current study demonstrates real-world evidence of a longer survival of advanced or recurrent GIST patients in the era of TKIs and identifies mutational status as a prognostic factor for survival of these patients. Other novel agents are under investigation and are expected to prolong the survival of advanced GIST patients in the near future.

### Supplementary Information

Supplementary Material 1.

Supplementary Material 2.

Supplementary Material 3.

Supplementary Material 4.

Supplementary Material 5.

Supplementary Material 6.

Supplementary Material 7.

Supplementary Material 8.

Supplementary Material 9.

## Data Availability

All data generated or analyzed during the current study are included in this published article and its supplementary information files. The DNA mutations identified in this study has been deposited to ClinVar with accession numbers SCV005061447—SCV005061477.

## Abbreviations

GISTs: Gastrointestinal stromal tumors
TKIs: Tyrosine kinase inhibitors
PFS: Progression-free survival
OS: Overall survival
CI: Confidence interval
ECOG PS: Eastern Cooperative Oncology Group performance status
NLR: Neutrophil to lymphocyte ratio
HR: Hazard ratio
FDA: Food and Drug Administration
