# Supplementary figures and images for: Survival of advanced/recurrent gastrointestinal stromal tumors treated with tyrosine kinase inhibitors in Taiwan: a nationwide registry study
Source: BMC Cancer. 2024 Jul 11;24:828. doi: 10.1186/s12885-024-12567-1 (PMC11238460; doi:10.1186/s12885-024-12567-1)

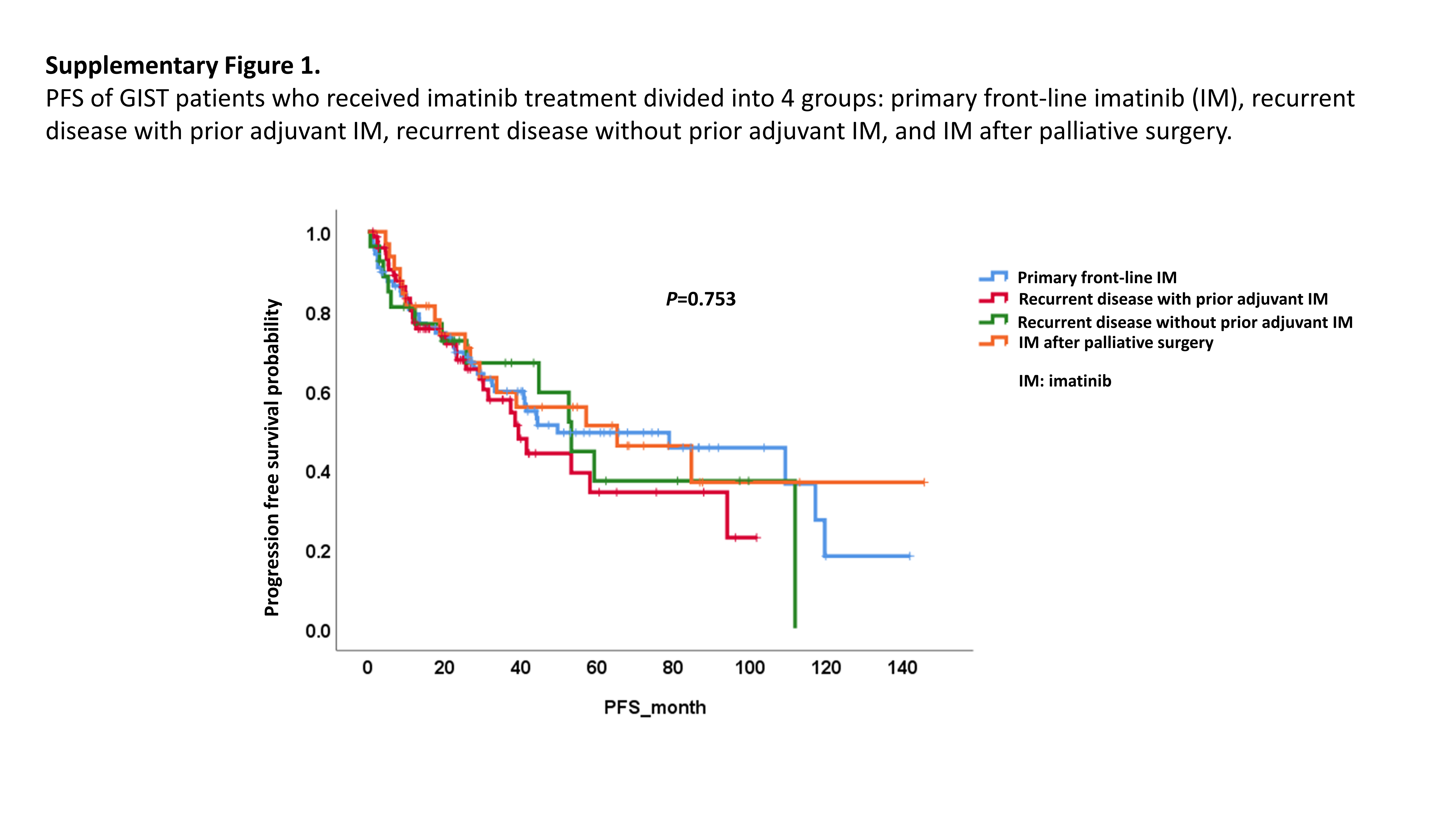

Supplement: Supplementary file 1 — Supplementary Material 1. [file 12885_2024_12567_MOESM1_ESM.tif]

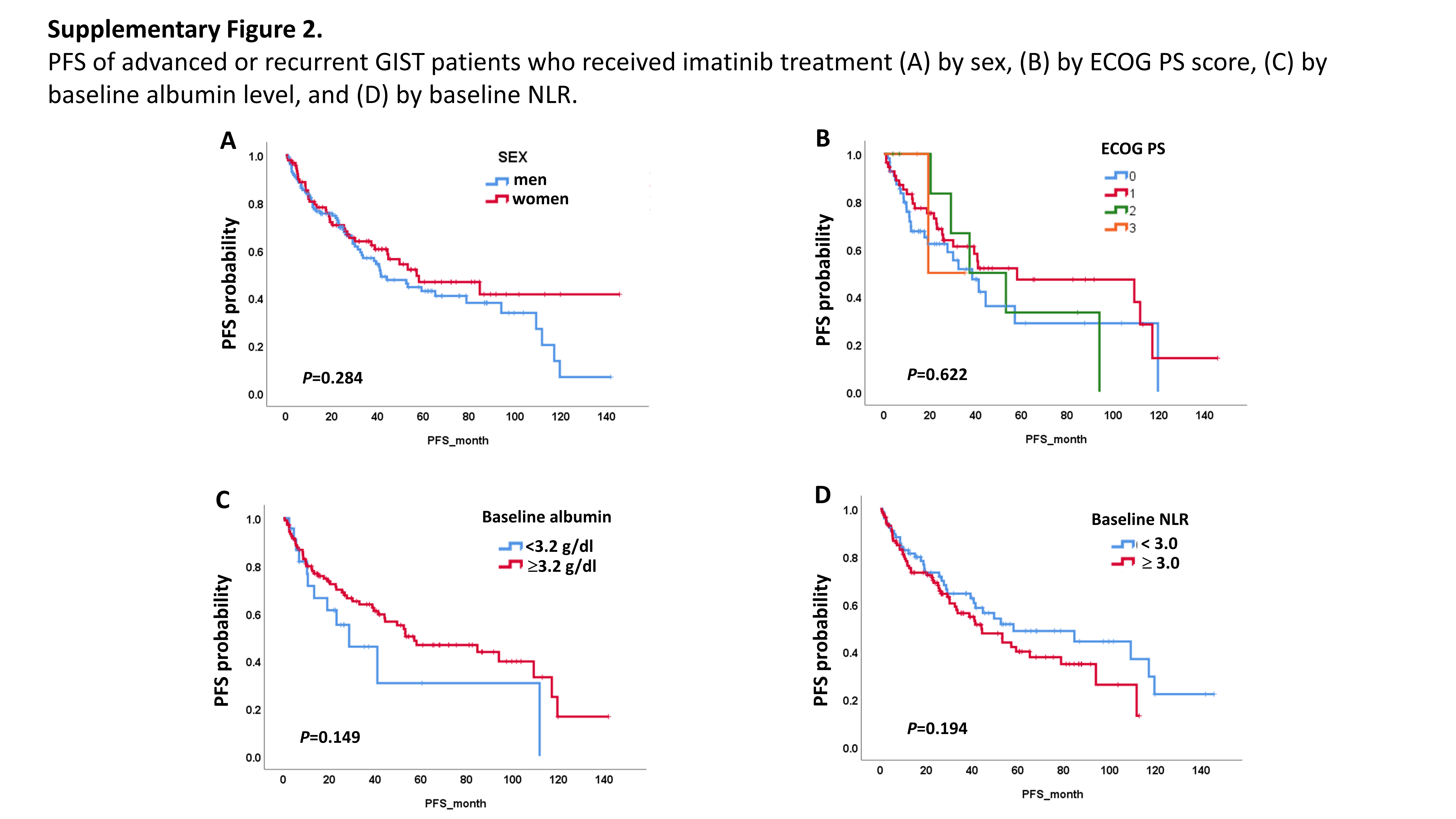

Supplement: Supplementary file 2 — Supplementary Material 2. [file 12885_2024_12567_MOESM2_ESM.tif]

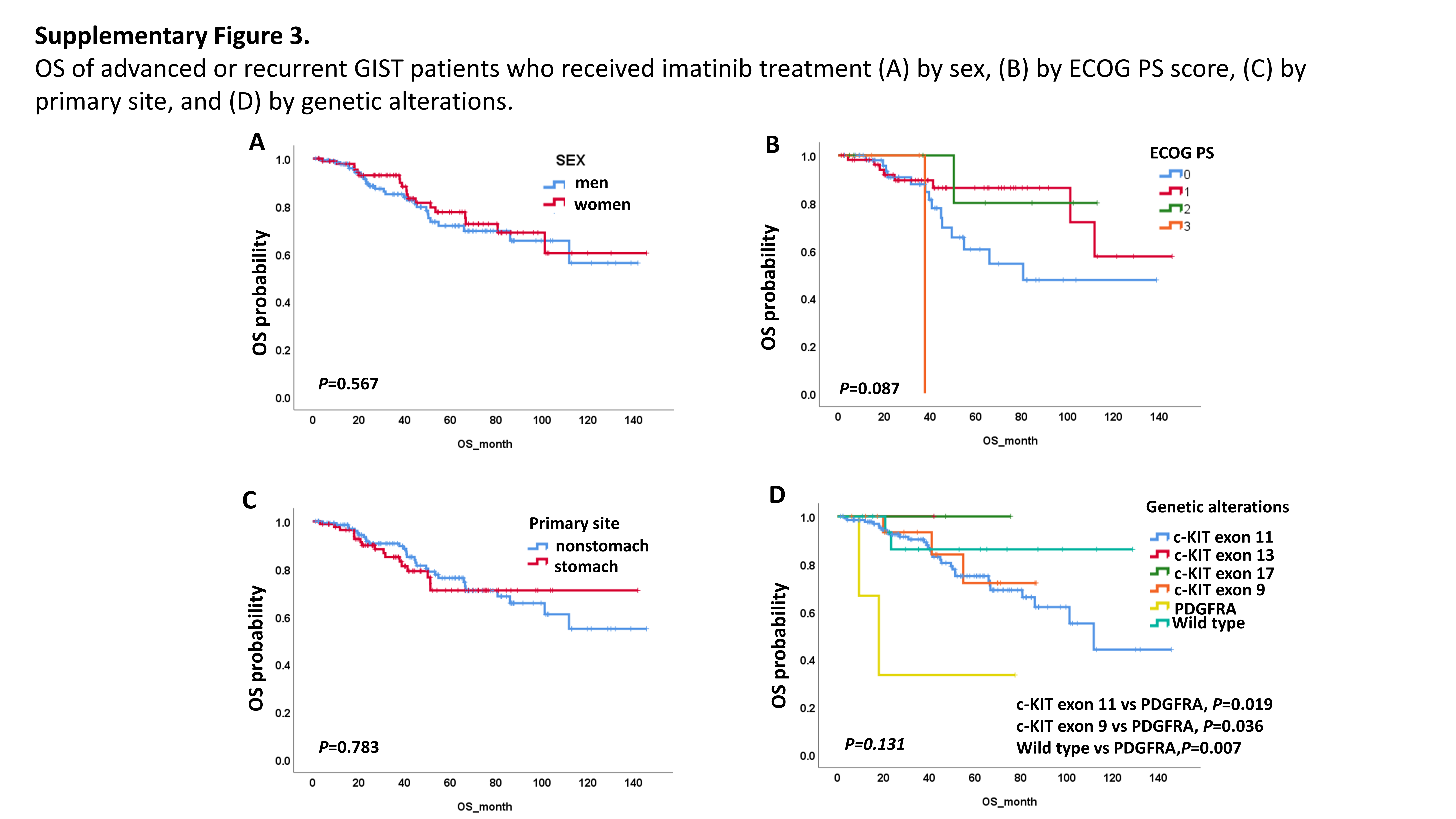

Supplement: Supplementary file 3 — Supplementary Material 3. [file 12885_2024_12567_MOESM3_ESM.tif]

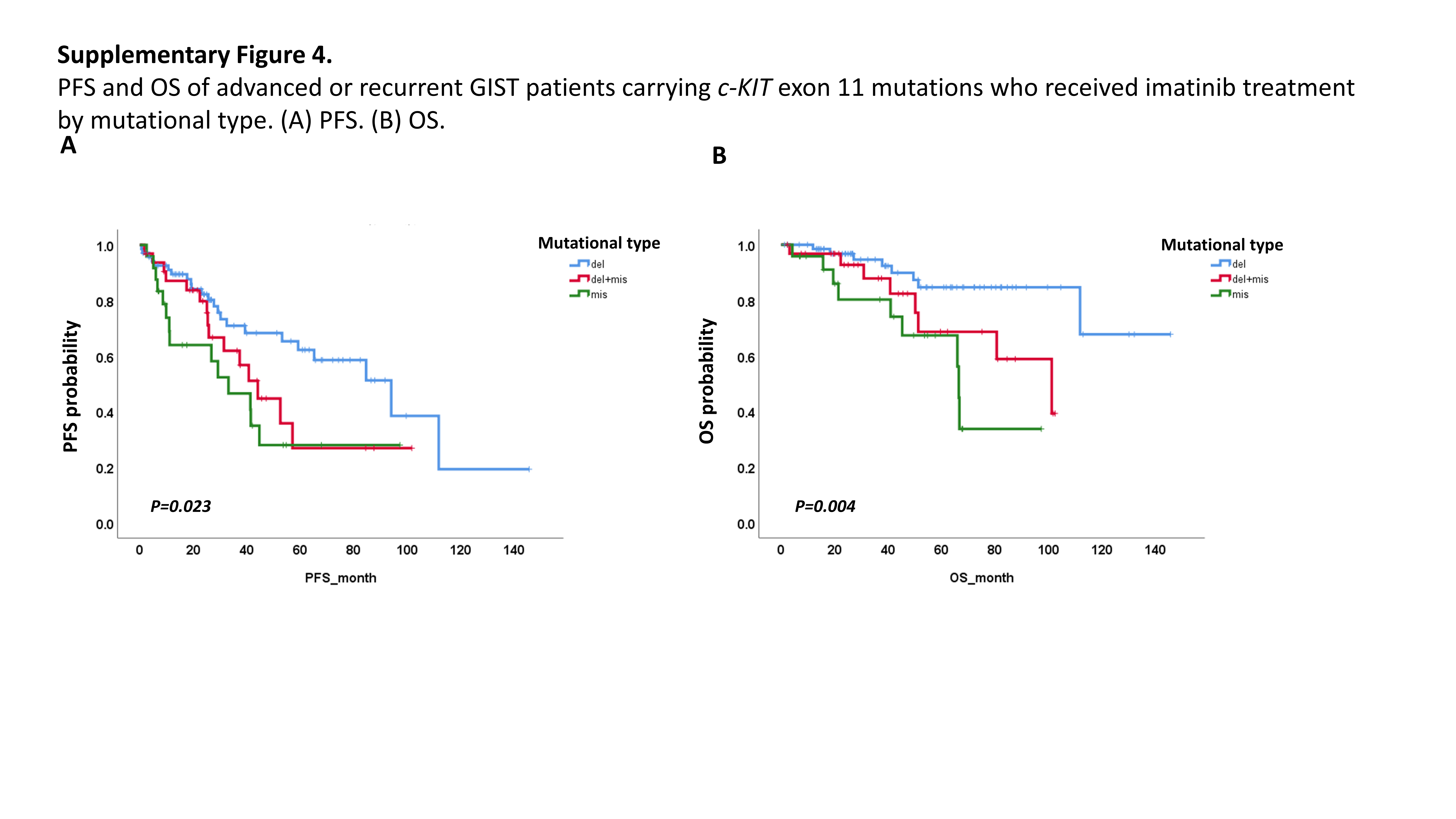

Supplement: Supplementary file 4 — Supplementary Material 4. [file 12885_2024_12567_MOESM4_ESM.tif]

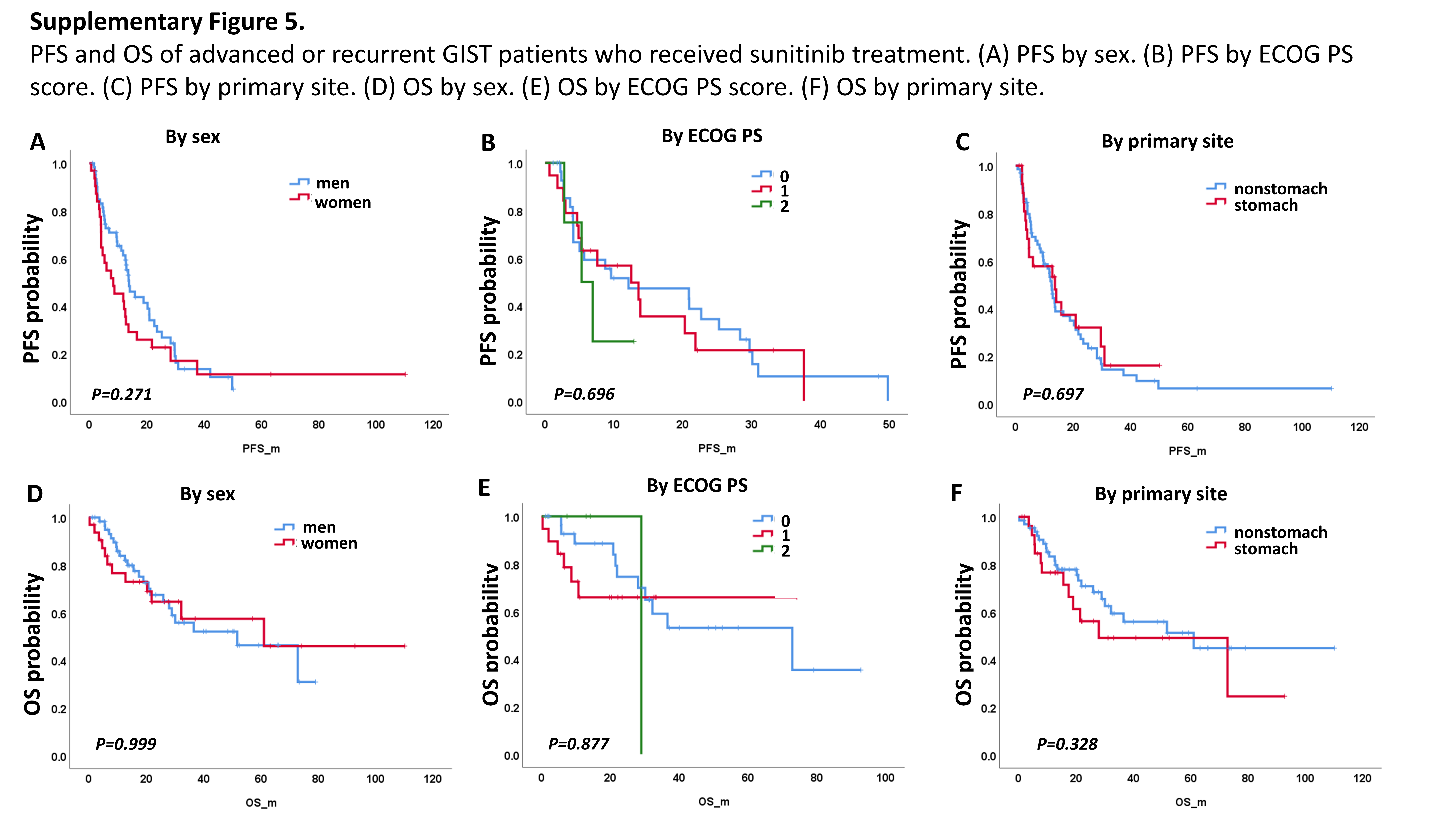

Supplement: Supplementary file 5 — Supplementary Material 5. [file 12885_2024_12567_MOESM5_ESM.tif]

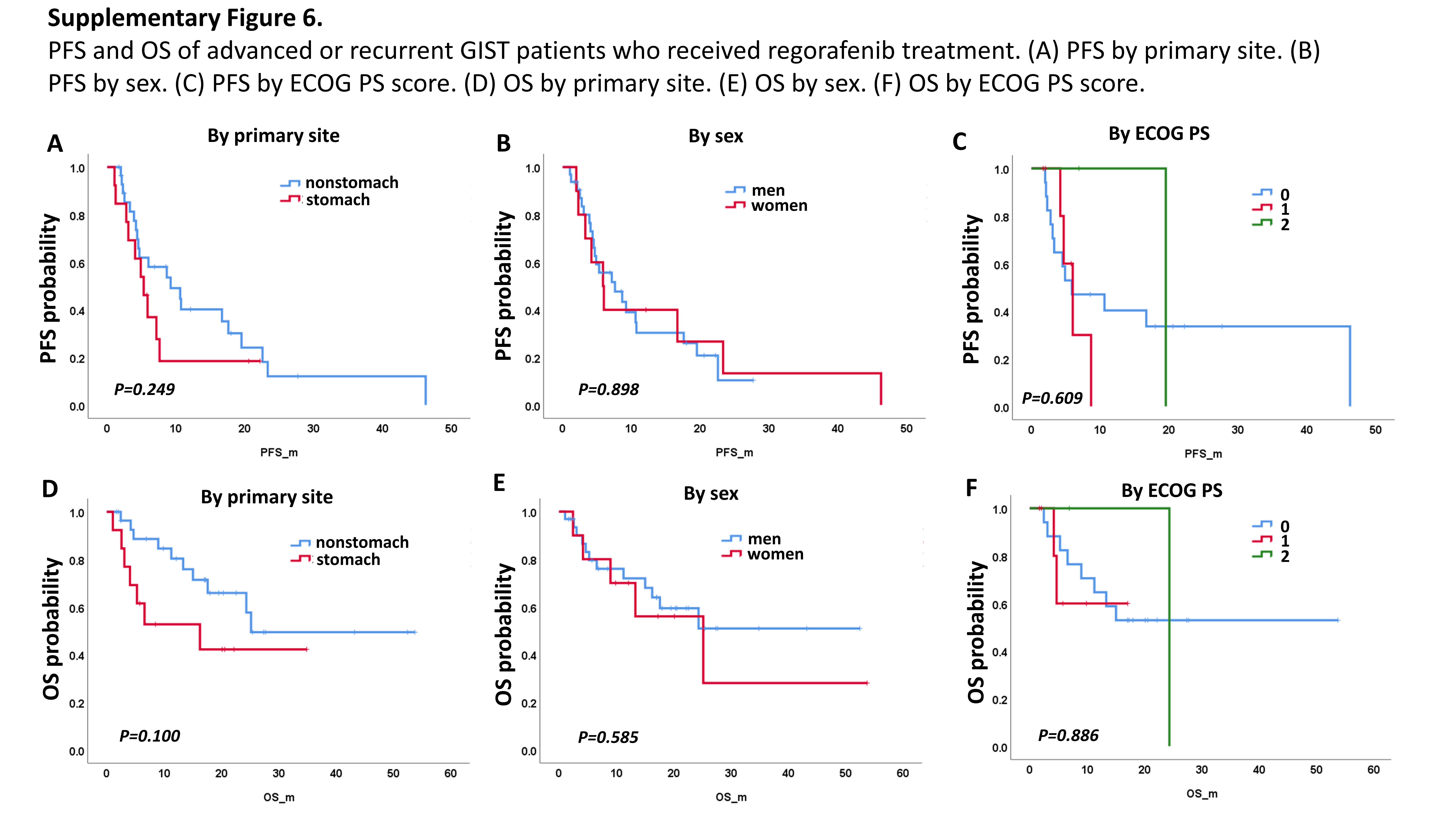

Supplement: Supplementary file 6 — Supplementary Material 6. [file 12885_2024_12567_MOESM6_ESM.tif]

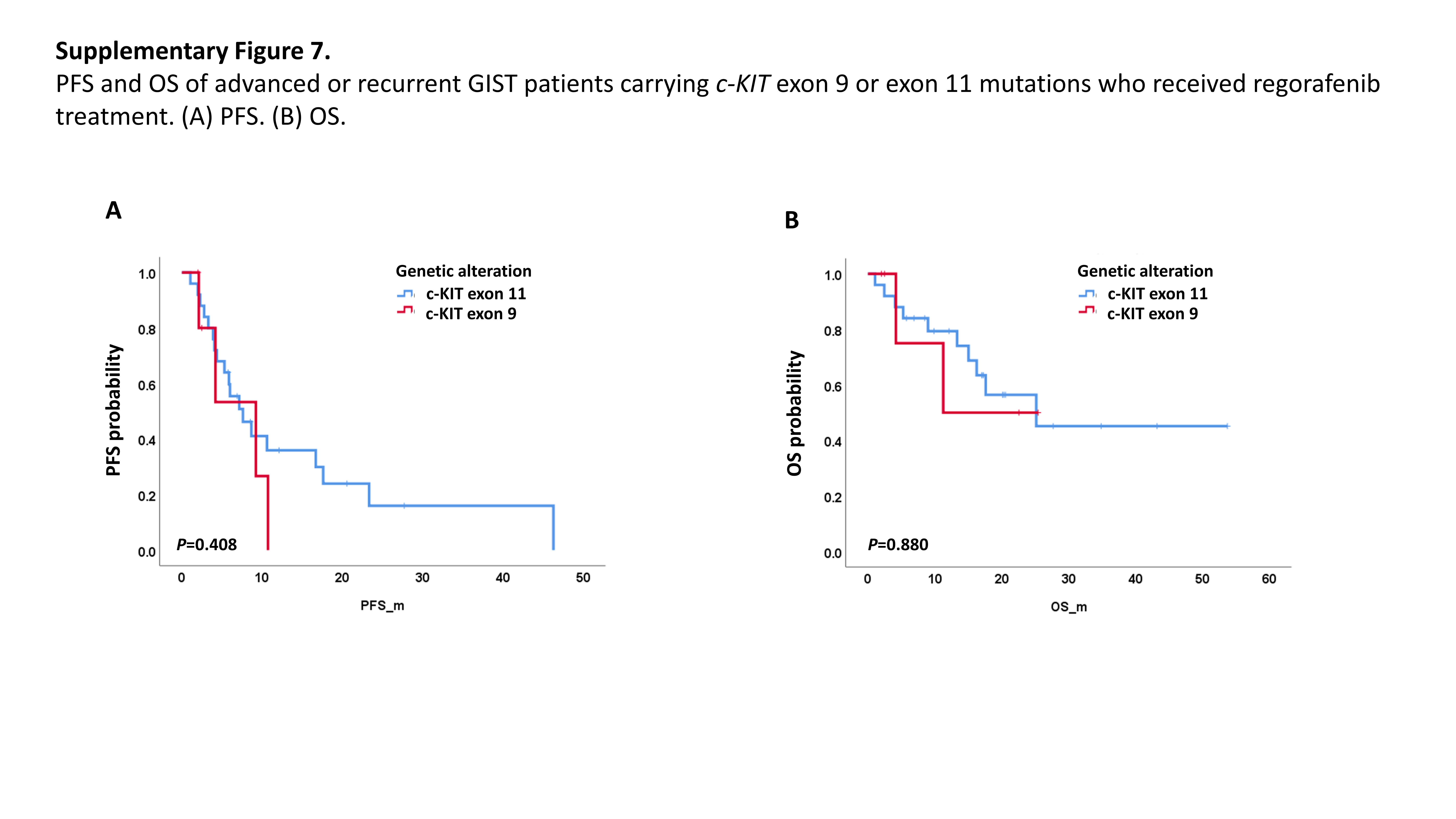

Supplement: Supplementary file 7 — Supplementary Material 7. [file 12885_2024_12567_MOESM7_ESM.tif]
