# Supplementary material for: Survival of advanced/recurrent gastrointestinal stromal tumors treated with tyrosine kinase inhibitors in Taiwan: a nationwide registry study
Source: BMC Cancer. 2024 Jul 11;24:828. doi: 10.1186/s12885-024-12567-1 (PMC11238460; doi:10.1186/s12885-024-12567-1)
Supplement: Supplementary file 8 — Supplementary Material 8. [file 12885_2024_12567_MOESM8_ESM.docx]

**Supplementary Method**

**DNA extraction**

Five sections of paraffin-embedded tissues of 10-µm thickness were used for DNA extraction for each patient. DNA was extracted by FavorPrep™ FFPE Tissue DNA Extraction Micro Kit (Favorgen, Ping Tung, Taiwan) according to the manufacturer's instructions. DNA concentration was determined using a NanoDrop 2000 spectrophotometer (Thermo Fisher Scientific, Waltham, MA, USA) to evaluate the quantity and quality of extracted DNA. The extracted DNA was stored at -20°C until use.

**Touchdown PCR amplification and sequencing for *c-KIT* and *PDGFRA* gene mutations**

PCR reaction mixture was 20 µL, which contained 18 μl of reaction mixtures (PCR Master Mix, each primer, and sterile deionized water), 1 μl of Taq DNA polymerase and 1 μl of DNA template (30-50 ng/μl). The following thermal conditions of touchdown PCR were applied: 94°C for 5 min; 94°C for 30 sec; 70°C - 53°C for 30 sec (-0.5°C per cycle); 72°C for 30 sec; then 30 cycles of denaturation at 94°C for 30 sec, annealing at 55°C for 30 sec, extension at 72°C for 30 sec and a final extension at 72°C for 5 min. Then, the samples were cooled down to 12°C for 10 min. The PCR products were used to detect the sequences of indicated exons for mutational analyses on exons 9, 11, 13, 14, and 17 in *c-KIT* and exons 12, 14, and 18 in *PDGFRA*. Primers sequences are shown in the following table.

**DNA Sequencing**

DNA sequencing was performed by Genomics Center for Clinical and Biotechnological Applications of National Core Facility for Biopharmaceuticals, Taiwan, which was supported by the Ministry of Science and Technology, MOST 110-2740-B-A49A-501.

**Table .**Primer sequences for *c-KIT* and *PDGFRA* exons

| **Primer** | **Sequence** |
| --- | --- |
| *c-KIT* |  |
| Exon 9 Forward | 5′-TCCTAGAGTAAGCCAGG-3′ |
| Exon 9 Reverse | 5′-CCCCTTAAATTGGATTAA-3′ |
| Exon 11 Forward | 5′-CATCAGTTTGCCAGTTGTGC-3′ |
| Exon 11 Reverse | 5′-AGCCCCTGTTTCATACT-3′ |
| Exon 13 Forward | 5′-CATCAGTTTGCCAGTTGT-3′ |
| Exon 13 Reverse | 5′-ACACGGCTTTACCTCCAATG-3′ |
| Exon 14 Forward | 5′-TGACCACCCTTGGGTATTTT-3′ |
| Exon 14 Reverse | 5′-CAGGAAGACTCCTTTGAATGC-3′ |
| Exon 17 Forward | 5′-TACAAGTTAAAATGAATTTAAATGGT-3′ |
| Exon 17 Reverse | 5′-AAGTTGAAACTAAAAATCCTTTGC-3′ |
| *PDGFRA* |  |
| Exon 12 Forward | 5′-AAGCTCTGGTGCACTGGGACTT-3′ |
| Exon 12 Reverse | 5′-GCAAGGGAAAAGGGAGTCTT-3′ |
| Exon 14 Forward | 5′-TGGTAGCTCAGCTGGACTGAT-3′ |
| Exon 14 Reverse | 5′-AATCCTCACTCCAGGTCAGT-3′ |
| Exon 18 Forward | 5′-ACCATGGATCAGCCAGTCTT-3′ |
| Exon 18 Reverse | 5′-TGAAGGAGGATGAGCCTGACC-3′ |
