# Supplementary material for: Survival of advanced/recurrent gastrointestinal stromal tumors treated with tyrosine kinase inhibitors in Taiwan: a nationwide registry study
Source: BMC Cancer. 2024 Jul 11;24:828. doi: 10.1186/s12885-024-12567-1 (PMC11238460; doi:10.1186/s12885-024-12567-1)
Supplement: Supplementary file 9 — Supplementary Material 9. [file 12885_2024_12567_MOESM9_ESM.docx]

Supplementary Table 1. Univariate Cox regression analysis for PFS of recurrent or metastatic GIST patients treated with imatinib treatment

| Univariate analysis | | | |
| --- | --- | --- | --- |
|  | HR | 95% CI | *P* value |
| Genetic alteration, referent *c-KIT* exon 11 | | | |
| *c-KIT* exon 13 | - | - | - |
| *c-KIT* exon 17 | 0.856 | 0.118-6.195 | 0.8780 |
| *c-KIT* exon 9 | 3.057 | 1.694-5.519 | 0.0002 |
| *PDGFRA* | 13.178 | 3.015-57.597 | 0.0006 |
| Wild-type *c-KIT/PDGFRA* | 0.887 | 0.417-1.886 | 0.7555 |
| Sex, referent men | | | |
| women | 0.805 | 0.541-1.198 | 0.2854 |
| ECOG PS, referent 0 | | | |
| 1 | 0.697 | 0.404-1.205 | 0.1962 |
| 2 | 0.920 | 0.352-2.408 | 0.8654 |
| 3 | 0.698 | 0.094-5.161 | 0.7248 |
| Age, referent < 60 y/o | | | |
| ≥ 60y/o | 0.836 | 0.561-1.244 | 0.3767 |
| Primary site, referent nonstomach | | | |
| stomach | 0.617 | 0.401-0.949 | 0.0280 |
| Baseline albumin level, referent < 3.2 g/dl | | | |
| ≥ 3.2 g/dl | 0.627 | 0.330-1.189 | 0.1524 |
| Baseline neutrophil/lymphocyte ratio, referent < 3.0 | | | |
| ≥ 3.0 | 1.332 | 0.863-2.055 | 0.1956 |

Supplementary Table 2. Univariate Cox regression analysis for OS of recurrent or advanced/metastatic GIST patients treated with imatinib treatment

| Univariate analysis | | | |
| --- | --- | --- | --- |
|  | HR | 95% CI | *P* value |
| Genetic alteration, referent *c-KIT* exon 11 | | | |
| *c-KIT* exon 13 | - | - | - |
| *c-KIT* exon 17 | - | - | - |
| *c-KIT* exon 9 | 0.815 | 0.248-2.678 | 0.7357 |
| *PDGFRA* | 4.815 | 1.140-20.345 | 0.0325 |
| Wild-type *c-KIT/PDGFRA* | 0.457 | 0.471-1.512 | 0.5679 |
| Sex, referent men | | | |
| women | 0.844 | 0.471-1.512 | 0.5679 |
| ECOG PS, referent 0 | | | |
| 1 | 0.441 | 0.183-1.065 | 0.0687 |
| 2 | 0.287 | 0.037-2.213 | 0.2308 |
| 3 | 2.932 | 0.367-23.422 | 0.3103 |
| Age, referent < 60 y/o | | | |
| ≥ 60y/o | 2.074 | 1.164-3.694 | 0.0133 |
| Primary site, referent nonstomach | | | |
| stomach | 1.088 | 0.597-1.983 | 0.7827 |
| Baseline albumin level, referent < 3.2 g/dl | | | |
| ≥ 3.2 g/dl | 0.365 | 0.158-0.841 | 0.0180 |
| Baseline neutrophil/lymphocyte ratio, referent < 3.0 | | | |
| ≥ 3.0 | 1.987 | 1.014-3.895 | 0.0454 |

Supplementary Table 3. Multivariate Cox regression analysis for OS of recurrent or advanced/metastatic GIST patients treated with imatinib treatment

| Univariate analysis | | | |
| --- | --- | --- | --- |
|  | HR | 95% CI | *P* value |
| Genetic alteration, referent *c-KIT* exon 11 | | | |
| *c-KIT* exon 13 | - | - | - |
| *c-KIT* exon 17 | - | - | - |
| *c-KIT* exon 9 | 0.804 | 0.167-3.861 | 0.7852 |
| *PDGFRA* | 98.670 | 5.200-1872.32 | 0.0022 |
| Wild-type *c-KIT/PDGFRA* | 0.354 | 0.046-2.737 | 0.3195 |
| Age, referent < 60 y/o | | | |
| ≥ 60y/o | 1.549 | 0.574-4.183 | 0.3880 |
| Baseline albumin level, referent < 3.2 g/dl | | | |
| ≥ 3.2 g/dl | 0.722 | 0.221-2.359 | 0.5898 |
| Baseline neutrophil/lymphocyte ratio, referent < 3.0 | | | |
| ≥ 3.0 | 1.785 | 0.604-5.273 | 0.2947 |

Supplementary Table 4. The pattern of mutational type in the recurrent or metastatic GIST patients with *c-KIT* exon 11 mutations.

|  | stomach | nonstomach | overall | *P** value |
| --- | --- | --- | --- | --- |
| Type of exon 11 mutation | 55 | 81 | 136 | 0.2263 |
| deletion | 27 (49.1%) | 42 (51.9%) | 69 (50.7%) |  |
| missense mutation | 6 (10.6%) | 18 (22.2%) | 24 (17.6%) |  |
| deletion+missense mutation | 15 (27.3%) | 17 (21.0%) | 32 (23.5%) |  |
| Deletion and insertion (delins) | 5 (9.1%) | 3 (3.7%) | 8 (5.9%) |  |
| duplication | 1 (1.8%) | 1 (1.2%) | 2 (1.5%) |  |
| unknown | 1 (1.8%) | 0 | 1 (0.7%) |  |

*, Fisher’s exact test

Supplementary Table 5. The baseline characteristics of the patients treated with sunitinib

|  | stomach | nonstomach | overall | *P** value |
| --- | --- | --- | --- | --- |
| Sex | 28 | 65 | 93 | 0.2422 |
| men | 21 (75.0%) | 40 (61.5%) | 61 (65.6%) |  |
| women | 7 (25.0%) | 25 (38.5%) | 32 (34.4%) |  |
| Diagnosed age | 28 | 65 |  |  |
| median age | 59.0 | 54.2 | 56.5 |  |
| range | 34.6-81.7 | 19.6-83.4 | 19.6-83.4 |  |
| mean | 58.8 | 55.3 | 56.4 |  |
| std | 9.9 | 13.5 | 12.6 |  |
| ECOG PS | 14 | 39 | 53 | 0.7026 |
| 0 | 9 (64.3%) | 21 (53.8%) | 30 (56.6%) |  |
| 1 | 5 (35.7%) | 14 (35.9%) | 19 (35.8%) |  |
| 2 | 0 | 4 (10.3%) | 4 (7.5%) |  |
| genetic data | 19 | 50 | 69 | 0.0017 |
| *c-KIT* exon 9 | 1 (5.3%) | 10 (20.0%) | 11 (76.8%) |  |
| *c-KIT* exon 11 | 13 (68.4%) | 40 (80.0%) | 53 (15.9%) |  |
| *PDGFRA* | 1 (5.3%) | 0 | 1 (1.4%) |  |
| Wild-type *c-KIT/PDGFRA* | 4 (21.1%) | 0 | 4 (5.8%) |  |

*, Fisher’s exact test.
